# Double diabetes—when type 1 diabetes meets type 2 diabetes: definition, pathogenesis and recognition

**DOI:** 10.1186/s12933-024-02145-x

**Published:** 2024-02-10

**Authors:** Weronika Bielka, Agnieszka Przezak, Piotr Molęda, Ewa Pius-Sadowska, Bogusław Machaliński

**Affiliations:** 1 Department of Diabetology and Internal Diseases, Pomeranian Medical University, 72-009 Police, Poland; 2 Department of General Pathology, Pomeranian Medical University, 70-111 Szczecin, Poland

**Keywords:** Diabetes, Type 1 diabetes, Type 2 diabetes, Double diabetes, Insulin resistance, Metabolic syndrome, Indirect insulin resistance markers

## Abstract

Currently, the differentiation between type 1 diabetes (T1D) and type 2 diabetes (T2D) is not straightforward, and the features of both types of diabetes coexist in one subject. This situation triggered the need to discriminate so-called double diabetes (DD), hybrid diabetes or type 1.5 diabetes, which is generally described as the presence of the insulin resistance characteristic of metabolic syndrome in individuals diagnosed with T1D. DD not only raises the question of proper classification of diabetes but is also associated with a significantly greater risk of developing micro- and macroangiopathic complications, which was independent of glycaemic control. When considering the global obesity pandemic and increasing incidence of T1D, the prevalence of DD may also presumably increase. Therefore, it is of the highest priority to discover the mechanisms underlying the development of DD and to identify appropriate methods to prevent or treat DD. In this article, we describe how the definition of double diabetes has changed over the years and how it is currently defined. We discuss the accuracy of including metabolic syndrome in the DD definition. We also present possible hypotheses connecting insulin resistance with T1D and propose possible methods to identify individuals with double diabetes based on indirect insulin resistance markers, which are easily assessed in everyday clinical practice. Moreover, we discuss adjuvant therapy which may be considered in double diabetic patients.

**Supplementary Information:**

The online version contains supplementary material available at 10.1186/s12933-024-02145-x.

## Introduction

According to the World Health Organisation (WHO), diabetes comprises a group of metabolic diseases characterised by chronic hyperglycaemia that may lead to damage in various organs (especially the eyes, kidneys, nerves, heart and blood vessels), which eventually results in dysfunction [1]. Two main types of diabetes (among others) have been distinguished: type 1 diabetes (T1D) and type 2 diabetes (T2D). This classification system is based on various factors that differ between patients with T1D and T2D, such as age at disease onset, excessive weight, degree of insulin resistance (IR), presence of metabolic syndrome (MS), degree of loss of pancreatic β-cell function, presence of specific autoantibodies associated with β-cell destruction, presence of a systematic subclinical inflammatory state, concentration of C-peptide in the blood and requirement for treatment with insulin to survive [2]. A patient suffering from T1D is typically represented as being a young lean person who has lost 90–100% of his/her β-cell function and needs insulin treatment from the time that the disease started because of a direct lack of insulin production and secretion. However, an individual with T2D is represented as being an older, overweight or obese person who develops the state known as insulin resistance, which causes the functional failure of β-cells and, consequently, insulin deficiency. Patients usually suffer from other diseases, especially cardiovascular diseases, and can be treated with oral antidiabetic drugs at the beginning of the course of the disease, whereas insulin treatment is needed at a later point in time.

However, at the current time, distinguishing between these two major types of diabetes is not straightforward, and the features of both T1D and T2D may coexist within one subject [3]. This state is known as double diabetes (DD), hybrid diabetes or type 1.5 diabetes and is generally described as the presence of the IR characteristic of metabolic syndrome in individuals diagnosed with type 1 diabetes [3, 4]. Merger et al. showed that one in four patients suffering from T1D meets the criteria of MS according to the National Cholesterol Education Program Adult Treatment Panel III (NCEP ATP III) and can be identified as an individual with DD [5]. This is associated with a significantly increased risk for the development of micro- and macroangiopathic complications (independent of glycaemic control) [5]. Moreover, the prevalence of cardiovascular comorbidities and diabetic nephropathy in individuals with DD is more similar to that in patients suffering from T2D than to that in patients with T1D [5]. This phenomenon creates new diagnostic and therapeutic challenges.

We do not currently possess global data on the prevalence of metabolic syndrome, and the estimates vary according to the criteria that are used to establish MS. However, given that MS is three times more common than diabetes, it can be estimated that metabolic syndrome affects approximately a quarter of the population worldwide [6]. A 2015 study highlighted the fact that 604 million adults and 108 million children in 195 countries were obese and that the prevalence of obesity doubled in more than 70 countries in individuals older than 25 years from 1980 to 2015 [7]. A trend towards an increasing incidence of excessive body weight has also been observed among patients suffering from T1D, most of whom are overweight or obese and gain weight faster than the general population [7, 8]. Of course, obesity is not always synonymous with the presence of MS but is usually one of its components and is easily diagnosed in most cases in everyday practice; thus, it should be particularly highlighted. When considering the global obesity pandemic and increasing incidence of type 1 diabetes, it is of the highest priority to establish the exact definition of double diabetes, as well as to discover the mechanisms underlying the development of DD and to identify appropriate methods to prevent or treat DD [7, 9].

In this article, we describe how the definition of double diabetes has changed over the years and how it is currently defined. We focused on the problems that could arise from the inclusion of the metabolic syndrome definition in the DD definition. We also present possible hypotheses connecting insulin resistance with type 1 diabetes. Moreover, we discuss the possible methods based on indirect insulin resistance markers for identifying individuals with double diabetes.

## Double diabetes—efforts to establish the classification criteria

The first mention of double diabetes was made in 1991. Teupe et al. showed that, compared with individuals without a family history of T2D, some patients with T1D who had relatives suffering from T2D have increased bodyweights and require higher doses of insulin to maintain normoglycaemia [3]. The authors proposed the classification of DD patients as type 1 diabetic individuals with at least one type 2 diabetic relative [3].

Moreover, paediatricians have reported an increased occurrence of T2D in children, which is unusual, as well as problems in differentiating between T1D and T2D in this population [4, 10–12]. Gilliam et al. defined DD patients as individuals who were atypical or who had clinical features characteristic of T1D and T2D at presentation, which raised doubts about the proper diagnosis [13]. Pozzilli et al. claimed that DD occurs in individuals with clinical features of T2D and T1D, with a metabolic component outweighing an autoimmune component [14]. Cleland also described DD as the presence of T1D and features characteristic of T2D [15]. Kietsiriroje et al. defined double diabetes as type 1 diabetes in individuals with a family history of T2D, being overweight, with features of MS or insulin resistance, thus highlighting the significance of an indirect marker of IR known as the estimated glucose disposal rate (eGDR) [16]. The double diabetes phenomenon may raise doubts as to whether there are more type 1 diabetes with coexisting features of type 2 diabetes or more type 2 diabetes with a present autoimmune component. It seems that DD represents a grey zone between T1D and T2D and is a continuum of diabetes [17]. A summary of DD definitions that have been created over the years is presented in Table 1.

**Table 1:** Summary of the efforts to define the classification criteria for double diabetes

Authors	Year	Diagnostic criteria for double diabetes	References
Teupe et al.	1991	• Diagnosed T1D • At least one relative suffering from T2D	[3]
Gilliam et al.	2005	• Atypical individuals or subjects with clinical features characteristic for both T1D and T2D at the presentation • Doubts about the proper diagnosis	[13]
Pozzilli et al.	2007	• Clinical features of T2D, such as: o Excessive body weight o Dyslipidaemia o Hypertension o Increased risk of cardiovascular diseases • Reduced number of typical clinical features of T1D, such as: o Polyuria o Polydipsia o Loss of weight o Ketoacidosis • Always obesity • Presence of autoantibodies against pancreatic β-cells in a reduced number and titre • Presence of family history of T1D or T2D (but not necessarily always present)	[14]
Cleland	2012	• Diagnosed T1D • Features characteristic for T2D, such as: o Relatively high doses of insulin needed to maintain appropriate glucose levels o Weight gain during insulin treatment o Presence of hypertension o Presence of IR (understood as low eGDR) o Low concentration of HDL-C o Family history of T2D, especially in at least 2 relatives	[15]
Kietsiriroje et al.	2019	• Diagnosed T1D • Family history of T2D • Presence of overweight, metabolic syndrome • Clinical features of insulin resistance – especially eGDR < 8	[16]

Descriptions in the text above. *eGDR* estimated glucose disposal rate, *HDL-C* high-density lipoprotein cholesterol, *IR* insulin resistance, *T1D* type 1 diabetes, *T2D* type 2 diabetes

Almost all of these authors focused on subjects with established T1D with features characteristic of T2D. The diagnostic criteria show one similarity involving elements of MS. Therefore, a type 1 diabetic individual with a diagnosis of metabolic syndrome may be classified as being a double diabetic individual. However, more precisely, insulin resistance underlies the pathogenesis of double diabetes, as explained further in the article. Moreover, the inclusion of MS in the criteria necessary to recognise DD may have some complications, thus making the diagnosis of double diabetes incoherent. Therefore, clinicians should first focus on assessing the presence of insulin resistance.

## In the double diabetes definition, does metabolic syndrome truly make the diagnosis easier?

As the thorough assessment of the degree of insulin resistance in type 1 diabetic patients in clinical practice is difficult, the presence of metabolic syndrome features may be helpful for identifying individuals at risk for double diabetes. The definition of metabolic syndrome has changed over the years. The most commonly used classification criteria are those proposed by the WHO, NCEP ATP III and the International Diabetes Federation (IDF), and modified later by (among others) IDF and American Heart Association (AHA) [18–23]. These data are presented in Table 2.

**Table 2:** The definitions of metabolic syndrome were established according to the WHO, NCEP ATP III and IDF

Criteria of metabolic syndrome		Organisation (year) (reference)			
		WHO (1999) [18, 19]	NCEP ATP III (2001, revised in 2005) [20, 21]	IDF (2005) [22]	Harmonised criteria provided, among others, by IDF and AHA (2009) [23]
Required		At least 1 of the following:	Not applicable	- Increased WC—values depending on ethnicity (for instance in Europeans ≥ 94 cm [men] or ≥ 80 cm [women]);—BMI > 30 kg/m^2^ is also acceptable	Not applicable
		- Impaired fasting glucose - Impaired glucose tolerance - Diabetes - IR (assessed by the HEC)			
Additional		AND at least 2 of the following:	Any 3 of the following:	AND any 2 of the following:	Any 3 of the following:
	Central obesity	- WHR > 0.9 [men] or > 0.85 [women] *and/or—*BMI > 30 kg/m^2^	- WC ≥ 102 cm (≥ 40 in) [men] or ≥ 88 cm (≥ 35 in) [women]	Included in the required criterion	- Increased WC—values depending on ethnicity (for instance in Europeans ≥ 94 cm [men] or ≥ 80 cm [women])
	HDL-C	- HDL-C < 0.9 mmol/L (< 35 mg/dL) [men] or < 1.0 mmol/L (< 39 mg/dL) [women] *and/or—*TG ≥ 1.7 mmol/L (≥ 150 mg/dL)	- < 1.03 mmol/L (< 40 mg/dL) [men] or < 1.29 mmol/L (< 50 mg/dL) [women] *or—*pharmacologic treatment for reduced HDL-C	- < 1.03 mmol/L (< 40 mg/dL) [men] or < 1.29 mmol/L (< 50 mg/dL) [women] *or—*pharmacologic treatment for reduced HDL-C	- < 1.03 mmol/L (< 40 mg/dL) [men] or < 1.29 mmol/L (< 50 mg/dL) [women] *or—*pharmacologic treatment for reduced HDL-C
	TG		- ≥ 1.7 mmol/L (≥ 150 mg/dL) *or—*pharmacologic treatment for elevated TG	- ≥ 1.7 mmol/L (≥ 150 mg/dL) *or—*pharmacologic treatment for elevated TG	- ≥ 1.7 mmol/L (≥ 150 mg/dL) *or—*pharmacologic treatment for elevated TG
	Glycaemia	Included in the required criterion	- Fasting glycaemia ≥ 5.6 mmol/L (≥ 100 mg/dL) *or—*pharmacologic treatment for hyperglycaemia	- Fasting glycaemia ≥ 5.6 mmol/L (≥ 100 mg/dL) *or—*diagnosed T2D	- Fasting glycaemia ≥ 5.6 mmol/L (≥ 100 mg/dL) *or—*pharmacologic treatment for hyperglycaemia
	Blood pressure	- BP ≥ 140/90 mmHg	- Systolic BP ≥ 130 mmHg *or—*diastolic BP ≥ 85 mmHg *or—*pharmacologic treatment for hypertension	- Systolic BP ≥ 130 mmHg *or—*diastolic BP ≥ 85 mmHg *or—*pharmacologic treatment for hypertension	- Systolic BP ≥ 130 mmHg *and/or—*diastolic BP ≥ 85 mmHg *or *pharmacologic treatment for hypertension
	Other	- Urinary albumin excretion rate 20 µg/min *or—*ACR ≥ 30 mg/g	–	–	–

*ACR* albumin-to-creatinine ratio, *AHA* American Heart Association, *BMI* body mass index, *BP* blood pressure, *HDL-C* HDL-cholesterol, *HEC* the hyperinsulinaemic-euglycaemic clamp, *IDF* International Diabetes Federation, *IR* insulin resistance, *NCEP ATP III* National Cholesterol Education Program Adult Treatment Panel III, *T2D* type 2 diabetes, *TG* triglyceride, *WC* waist circumference, *WHO* World Health Organisation, *WHR* waist-to-hip ratio

The MS criteria are based on uncomplicated measurements and basic blood tests; however, all of them involve hyperglycaemia or diagnosed diabetes, which are intended to be related to IR and T2D. However, type 1 diabetic individuals will always meet these criteria, but not necessarily because of the development of insulin resistance. Moreover, patients may also be diagnosed with MS according to the WHO definition when they only possess microalbuminura, even if they have a normal body weight and lipid profile. For this reason, the definitions of metabolic syndrome should be used carefully in these patients so that they are not overdiagnosed with MS. This also raises the question of whether this glycaemic criterion could be supported by or replaced with other markers indicating the presence of insulin resistance in individuals with type 1 diabetes. This problem was addressed by Lecumberri et al. They showed that the prevalence of MS among type 1 diabetic patients is lower when using standard IDF criteria and when excluding hyperglycaemia as a criterion [24]. Moreover, they proposed the modification of the MS definition in T1D, including the assessment of IR by using indirect insulin resistance markers [24]. Another disadvantage of using MS definitions is the lack of cohesion among them. These conditions are not the same, and some discrepancies in the epidemiology of metabolic syndrome may occur, as the prevalence of MS may vary depending on the criteria that are used while performing research [25–29]. Hence, special attention should be given when using MS definitions among individuals with type 1 diabetes.

Due to the fact that double diabetes is associated with a significantly greater risk of developing micro- and macroangiopathic complications (similar to T2D rather than T1D), the quick, appropriate and efficient identification of the population at risk for DD development is crucial [5]. The aforementioned issues indicate the need for more precise tools to diagnose double diabetes that focus on the presence of insulin resistance and not on the presence of metabolic syndrome itself, which may be unreliable and insufficient in double diabetes diagnosis.

## Insulin resistance and type 1 diabetes—possible explanations for this association

The pathophysiology of the coexistence of type 1 diabetes and insulin resistance is likely multifactorial and has not been fully established. First, type 1 diabetic subjects may also have a genetic predisposition to IR and T2D development, especially when they have a relative with diagnosed T2D [16]. This claim is supported by the fact that people with a family history of T2D tend to have a higher BMI and body fat percentage and exhibit decreased insulin sensitivity [3, 30]. It has been suggested that some type 1 diabetic individuals with a T2D family history develop T2D at some point in life if they have not developed T1D that is initially triggered by an independent pathological process [3]. This hypothesis points to an autoimmune process as a phenomenon that occurs accidentally and independently from insulin resistance and obesity in predisposed individuals.

In contrast, the accelerator hypothesis focuses on obesity-driven insulin resistance in at-risk individuals, which leads to glucotoxicity and accelerates the apoptosis of pancreatic β-cells [31, 32]. It increases immunogenicity and consequently leads to T1D occurrence [31, 32]. To a certain extent, the overload hypothesis is similar. In individuals with triggered autoimmune processes, excessive body weight leads to insulin resistance, which causes the overload of β-cells and the acceleration of apoptosis in these cells [33]. Both of these hypotheses are supported by research, in which insulin resistance and excessive body mass were proven to have an impact on T1D development by accelerating its onset and increasing its risk [34–36].

It is possible that insulin resistance develops later after T1D diagnosis due to treatment. Intensive insulin therapy reduces the risk of long-term diabetic complications but is also associated with weight gain [37–39]. Obesity is a well-known factor associated with IR that causes chronic inflammation [40, 41]. As previously mentioned, type 1 diabetic subjects gain weight faster than does the general population [8]. This is caused not only by intensive insulin treatment but also by cultural, social and lifestyle changes that favour unhealthy diets and sedentary lifestyles. More flexible insulin treatment adjusted to patients’ actual meals allows for a less cautious dietary pattern, such as an increased frequency of snack consumption. Additionally, repetitive episodes of hypoglycaemia induced by insulin therapy or the presence of fear of hypoglycaemia may result in maladaptive eating habits that promote weight gain [42, 43]. Moreover, obesity itself does not seem to be the only factor responsible for IR. Donga et al. demonstrated that insulin resistance is also present in lean individuals suffering from type 1 diabetes [44]. These findings suggest that the pathogenesis of IR in T1D patients cannot be solely explained by excessive body weight.

Moreover, treatment for T1D is not completely physiological. Normally, insulin is secreted from the pancreas into the portal vein and subsequently reaches the liver, where it is mostly metabolised. Conversely, during T1D treatment, insulin is absorbed from the subcutaneous tissue to the peripheral circulation, thus resulting in peripheral hyperinsulinaemia and hepatic hypoinsulinaemia. It has been suggested that adaptation to this condition may result in decreased peripheral glucose uptake mediated by insulin and increased glucose production by the liver [45]. Chronically elevated levels of insulin in peripheral tissues may modify the expression and activity of insulin receptors, thus influencing insulin signalling pathways and worsening the degree of insulin sensitivity in peripheral tissues [46]. Hyperinsulinaemia interferes with mitochondrial function and enhances oxidative stress in T1D mice, thus increasing whole-body and hepatic insulin resistance [47]. Aditionally, chronic hyperglycaemia increases IR by inducing glucotoxicity, which participates in reducing peripheral glucose uptake; moreover, intensive insulin therapy in T1D patients was shown to increase the degree of insulin sensitivity [48–50]. One of the available methods for treating T1D is continuous intraperitoneal insulin infusion, which has been shown to mimic the physiological route of insulin, as insulin reaches the liver first after diffusion (mainly via the portal vein) [51]. This results in the restoration of the natural gradient of insulin concentrations between the liver circulation and the peripheral circulation [52]. In contrast, a decrease in peripheral insulin concentration due to continuous intraperitoneal insulin infusion was not associated with an improvement in insulin resistance, as assessed by using the hyperinsulinaemic-euglycaemic clamp technique (HEC) [53]. This fact indicates that the presence of insulin resistance cannot be solely explained by the nonphysiological nature of type 1 diabetes treatment.

**Fig. 1:**
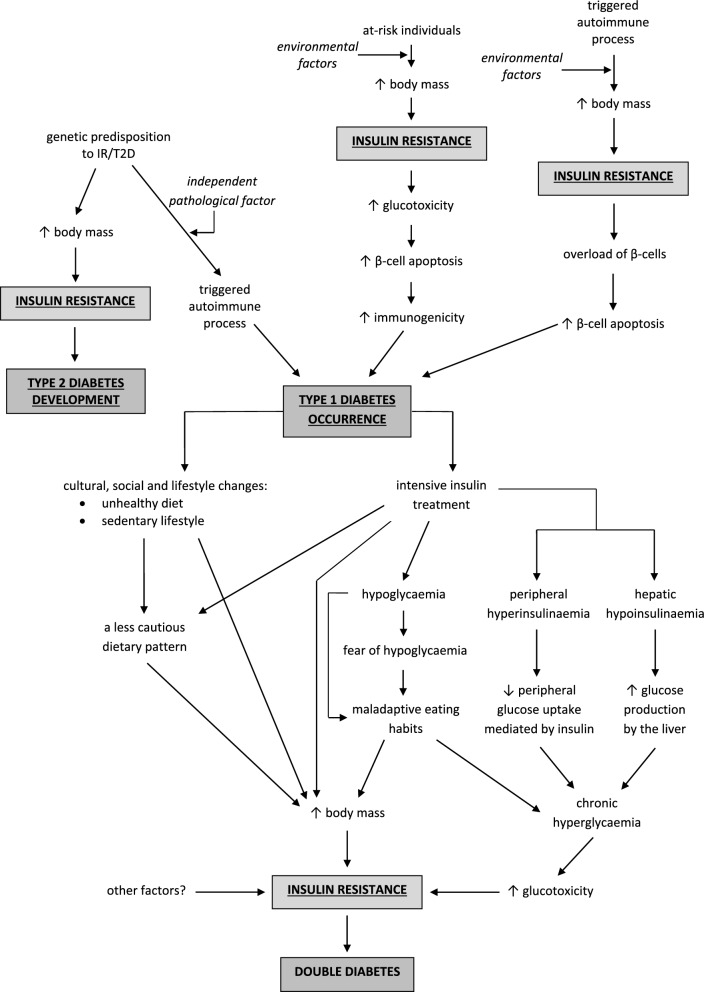
Title: Pathophysiology of double diabetes. Descriptions in the text above. IR, insulin resistance; T2D, type 2 diabetes

In 1986, Yki-Jarvinen et al. showed that most individuals with T1D of long duration are characterised by varying degrees of IR [54]. This was shown in both poorly controlled and adequately controlled type 1 diabetic patients [55, 56]. These findings were further confirmed in a meta-analysis performed by Donga et al. [57]. Therefore, the identification of tools for assessing the exact degree of insulin sensitivity in patients with type 1 diabetes and distinguishing between individuals with a “normal” level of insulin resistance in type 1 diabetic patients and those with a pathologically high level of insulin resistance are highly important. The pathophysiology of double diabetes is presented in Fig. 1.

## Recognition of double diabetes—the role of indirect insulin resistance markers

The gold standard for measuring insulin-mediated glucose uptake is still the hyperinsulinaemic-euglycaemic clamp technique, in which the glucose disposal rate is obtained (GDR) [58]. However, this method is relatively invasive, expensive, time-consuming and technically complicated; as a result, it is not used in everyday clinical practice. The homeostatic model assessment for insulin resistance (HOMA-IR) is typically used for routine assessment of insulin resistance [59]. It has been acknowledged to be a useful tool in epidemiology studies for assessing insulin sensitivity [60, 61]. However, the HOMA-IR cannot be used in patients suffering from type 1 diabetes, because the function of pancreatic β-cells is not known. Additional indirect insulin resistance indices, such as the quantitative insulin sensitivity check index (QUICKI) or the Matsuda index, have been established; however, most of these indices cannot be applied in type 1 diabetic patients [62, 63]. The assessment of insulin sensitivity by these aforementioned indices among individuals who are completely dependent on exogenous insulin is problematic, as fasting glycaemia and insulinaemia do not reflect insulin and glucose metabolism but rather the treatment itself. Therefore, further research must be performed to establish helpful markers indicating the presence of insulin resistance in patients with T1D and to consequently diagnose double diabetes properly.

An alternative method for assessing the presence of IR is to use indirect markers of insulin resistance based on widely available clinical parameters, such as medical history, anthropometrical parameters and basic laboratory tests. The first such marker was the estimated glucose disposal rate (eGDR) [64, 65]. As previously mentioned, a value of eGDR less than 8 was used in the classification criteria for double diabetes by Kietsiriroje et al. [16]. Other surrogate measures of insulin resistance include the lipid accumulation product (LAP), the triglyceride-glucose index (TyG-index) and the visceral adiposity index (VAI) [66–69]. In addition, other measures include parameters related to the TyG index, such as the triglyceride-glucose-body mass index (TyG-BMI) and the triglyceride-glucose-waist circumference (TyG-WC) [70]. Moreover, other parameters include the estimated insulin sensitivity (eIS), the natural logarithm of the glucose disposal rate (lnGDR), the metabolic score for IR (METS-IR), the triglyceride-glucose-waist-to-height ratio (TyG-WHtR) and the triglyceride-glucose-waist-to-hip ratio (TyG-WHpR) [71–75]. Furthermore, the insulin dosage that is needed for obtaining normoglycaemia per kg, waist-to-height ratio (WHtR), waist circumference (WC) and the triglyceride/HDL cholesterol ratio (TG/HDL-C) may also be used in the screening of metabolic syndrome among adult patients suffering from type 1 diabetes [76–79]. The aforementioned indirect insulin resistance markers are fast, inexpensive and noninvasive methods and may be useful tools for identifying IR in people for whom the HOMA-IR, QUICKI or Matsuda index cannot be applied. A summary of these data is presented in Additional file 1.

Most of these indirect markers of insulin resistance were compared with the gold standard (HEC) in different populations, which showed efficacy in assessing the presence of insulin resistance; these markers included eGDR, VAI, eIS, lnGDR, METS-IR, the TG-HDL-C ratio, the TyG index, the LAP and the TyG-BMI [64, 69, 71–73, 79, 94, 120, 121]. Most likely, the TyG index and related parameters (TyG-BMI, TyG-WC, TyG-WHpR, and TyG-WHtR) are not suitable for routine use in type 1 diabetic patients because they contain fasting plasma glucose in their formulas, which is strictly dependent on intensive insulin treatment; however, this hypothesis has not yet been confirmed. Perhaps a modification of their formulas and replacement of fasting glycaemia with other clinical values would be sufficient for use them in T1D patients.

The results of studies comparing indirect insulin resistance markers are incoherent and it is impossible to indicate at the moment which marker is better than others [71, 81, 122]. Moreover, the specific cut-points of different insulin resistance markers for IR and/or MS detection may vary depending on the ethnicity and the statistical methods used in studies. The existing differences were presented in Additional file 1, highlighting the need to be cautious while comparing studies.

## Treatment in double diabetes, can insulin succeed alone?

The only treatment for type 1 diabetes is insulin treatment (preferably as intensive insulin injections) and must not be changed into other antihyperglycaemic drugs. But intensive insulin treatment is associated with weight gain which escalates insulin resistance and leads to higher doses of insulin needed to achieve glycaemic targets [123]. Because of that, glucose-lowering drugs commonly used in therapy of type 2 diabetes (especially metformin, sodium-glucose co-transporter type 2 inhibitors and glucagon-like peptide-1 receptor agonists) can be promising possibilities in double diabetes. These medications are not officially indicated to be used among patients with T1D, but they may improve insulin sensitivity in these patients. Moreover, they can influence the components of metabolic syndrome, such as blood pressure or lipid profile.

Metformin decreases hepatic gluconeogenesis and increases peripheral glucose uptake stimulated by insulin through different, still not fully understood pathways [124]. A 2015 meta-analysis showed a significant reduction in weight and total daily dose of insulin when metformin was applied in T1D patients, but these positive effects vanished in the long-term observation [125, 126]. The REMOVAL trial suggested that metformin may have a wider role in cardiovascular risk management in patients with T1D [127]. Metformin addition to insulin therapy can also increase insulin sensitivity in type 1 diabetic individuals [128, 129]. Oza et al. suggested that metformin addition may prevent form deteriorating insulin sensitivity in type 1 diabetic adolescents and, consequently, prevent from the development of double diabetes [130]. The positive benefits of metformin were not associated with the improvement of glycaemic control, nor with the increased risk of hypoglycaemia or diabetic ketoacidosis (DKA) [125–127]. Metformin seems to be a reasonable choice in double diabetic individuals because of its ability to reduce insulin resistance, beneficial cardiovascular influence and its safety.

Sodium-glucose co-transporter type 2 (SGLT-2) inhibitors reduce reabsorption of filtered glucose in renal proximal tubules which causes glycosuria and lowers glycaemia. It is a group of drugs which attracts attention of many different specialists by its additional functions. Beneficial influence of SGLT-2 inhibitors on cardiovascular and kidney outcomes in patients with type 2 diabetes and in non-diabetic patients triggers the need to assess whether they could be used in T1D.The EASE trial, the DEPICT-1 and DEPICT-2 study investigated the use of empagliflozin and dapagliflozin, respectively, in type 1 diabetic patients [131, 132]. Both of the substances showed the reduction in HbA1c, body mass and daily dose of insulin but increased the risk of diabetic ketoacidosis [131, 132]. SGLT-2 inhibitors seem to be one of the possible therapies in double diabetes, especially in individuals with an excessive body weight and/or present cardiovascular risk factors, but the patients should be aware of the risk of diabetic ketoacidosis and should be able to recognise the symptoms of diabetic ketoacidosis and react properly by themselves.

Glucagon-like peptide-1 receptor agonists (GLP1-RAs) are incretin drugs which stimulate glucose-dependent insulin secretion, inhibit glucagon secretion from pancreatic α cells during hyperglycaemia, slow down the emptying of stomach and influence the appetite leading to the reduction in food intake [133]. The ADJUNCT trials showed that liraglutide administration reduced HbA1c, daily dose of insulin and body weight in type 1 diabetic patients, but increased the risk of hypoglycaemia and ketosis [134, 135]. GLP1-RAs should be considered when a patient has excessive body weight.

Lifestyle changes are also necessary to prevent and treat insulin sensitivity disorders in type 1 diabetic patients. Diet is an important modifiable risk factor of the development of insulin resistance in T1D, and diet with high protein, low fat and optimum carbohydrate intake with increased intake of dietary fiber may improve insulin sensitivity [136]. Dietary modifications, especially an isocaloric low-fat diet, may improve insulin sensitivity, which is not associated with weight loss or improvement in glycaemic control [137]. Regular physical activity can also be beneficial, as it reduces insulin resistance and daily dose of insulin without influencing HbA1c [138–140]. These lifestyle modifications should not be underestimated as an adjunctive treatment in double diabetes.

## Summary

Double diabetes, which is a situation in which two main types of diabetes exist in one patient, raises doubts about whether the current classification of diabetes is still in use. It is a serious clinical problem, as it is associated with a significantly increased risk of developing complications (especially macrovascular complications), independent of glycaemic control. Moreover, it usually requires additional interventions, such as lifestyle modification or the addition of metformin, SGLT-2 inhibitors or GLP-1RAs to insulin therapy. Validation of a proper definition of DD is necessary to recognise individuals who are at risk for or who have already developed this condition. The use of the definition of metabolic syndrome may be helpful in double diabetes diagnosis; however, its role should not be overestimated, as the lack of unified criteria and glycaemic criteria that are consistently present in type 1 diabetic individuals may cause some discrepancies and may be misleading. The assessment of the degree of insulin resistance should eliminate these problems arising from using the metabolic syndrome definition in DD diagnosis, especially as IR underlies the pathophysiology of double diabetes. This approach will likely make the diagnosis of DD more reliable and objective. Indirect insulin resistance markers deserve special attention because they can be helpful in assessing the severity of insulin sensitivity disorders, and they are based on easy, inexpensive, widely available clinical parameters, such as medical history, anthropometrical parameters and basic laboratory tests. Furthermore, they are easy to incorporate in everyday clinical practice and could be used in the future to both recognise double diabetes without doubt (after further studies are performed on the parameters to validate them) and to monitor responses to particular interventions.

### Supplementary Information

**Additional file 1.** Summary of indirect insulin resistance markers.

## Data Availability

Not applicable.

## Abbreviations

ACR: Albumin-to-creatinine ratio
AHA: American Heart Association
BMI: Body mass index
BP: Blood pressure
CV: Cardiovascular
DD: Double diabetes
DDI: Daily dose of insulin
eGDR: The estimated glucose disposal rate
eIS: The estimated insulin sensitivity
FPG: Fasting plasma glucose
GDR: Glucose disposal rate
GLP-1RAs: Glucagon-like peptide-1 receptor agonists
HbA1c/HbA1: Glycated haemoglobin
HDL-C: High-density lipoprotein cholesterol
HEC: Hyperinsulinaemic-euglycaemic clamp
HOMA-IR: The homeostatic model assessment for insulin resistance
HT: Hypertension
IDF: International Diabetes Federation
IR: Insulin resistance
LAP: The lipid accumulation product
lnGDR: The natural logarithm of the glucose disposal rate
METS-IR: The metabolic score for insulin resistance
MS: Metabolic syndrome
NCEP ATP III: National Cholesterol Education Program Adult Treatment Panel III
QUICKI: The quantitative insulin sensitivity check index
SGLT-2: Sodium-glucose co-transporter type 2
ST1RE: The Steno type 1 risk engine
T1D: Type 1 diabetes
T2D: Type 2 diabetes
TG: Triglyceride
TG/HDL-C ratio: The triglyceride/high-density lipoprotein cholesterol ratio
TyG index: The triglyceride-glucose index
TyG-BMI: Triglyceride-glucose-body mass index
TyG-WC: Triglyceride-glucose-waist circumference
TyG-WHpR: Triglyceride-glucose-waist-to-hip ratio
TyG-WHtR: Triglyceride-glucose-waist-to-height ratio
U: Unit
VAI: The visceral adiposity index
WC: Waist circumference
WHO: World Health Organisation
WHR = WHpR: Waist-to-hip ratio
WHtR: Waist-to-height ratio
