# Supplementary material for: Double diabetes—when type 1 diabetes meets type 2 diabetes: definition, pathogenesis and recognition
Source: Cardiovasc Diabetol. 2024 Feb 10;23:62. doi: 10.1186/s12933-024-02145-x (PMC10859035; doi:10.1186/s12933-024-02145-x)
Supplement: Supplementary file 1 — Additional file 1. Summary of indirect insulin resistance markers. [file 12933_2024_2145_MOESM1_ESM.pdf]

Additional file 1. Summary of indirect insulin resistance markers.

Indirect insulin resistance marker	Authors (reference)	Formula	Utility of assessing IR and/or MS in individuals with T1D	Cut-off point for IR and/or MS in adult individuals with T1D	Comment (with the highlight whether the statement was formed on the grounds of research performed in diabetic populations and, if so, in which type of diabetes)
eGDR	Williams et al. 2000 (64)	original formula $24.31 - (12.22 \times \mathrm{WHR}) - (3.29 \times \mathrm{HT}[0 - \mathrm{no}; 1 - \mathrm{yes}] - (0.57 \times \mathrm{HbA1}[\%])$	IR – (64,80)<br><br>MS – (80,81)	for IR assessment:<br><6.4 (82);<br><7.5, but without explanation why (83);<br>varies from 5 to 9 (82);<br><br>for MS assessment:<br><7.32 (81);<br><8.77 (80);<br><br>for DD assessment:<br><8.0 (16);	- T1D: lower values in IR (80)<br>- T1D: low values when diabetic retinopathy, neuropathy or nephropathy coexist (80)<br>- T1D: predicts the development of peripheral vascular disease, coronary artery disease and nephropathy (84)<br>- T1D: higher values independently associated with a lower risk for CV events (85)<br>- T1D: strongly associated with all-cause and CV mortality (84)<br>- T1D: one of the most sensitive methods to detect MS independently of gender (81)<br>- T1D: the cut-off point for predicting CV disease risk according to the ST1RE is <8.52 (moderate/high risk) and <8.08 (high risk) (86)<br>- the original formula contained HbA1 method, and not currently used HbA1c (64)
	Thorn et al. 2005 (65)	modified formula $24.40 - (12.97 \times \mathrm{WHR}) - (3.39 \times \mathrm{HT}[0 - \mathrm{no}; 1 - \mathrm{yes}] - (0.60 \times \mathrm{HbA1c}[\%])$			

					- modified formula contains HbA1c method (65)<br>- other formulas contain WC or BMI instead of WHR (64,84,87)
LAP	Kahn 2005 (66)	men<br>$(\mathrm{WC[cm]} - 65) \times \mathrm{TG}[\frac{\mathrm{mmol}}{\mathrm{L}}]$<br>if WC≤65.0 cm, assume WC=66.0 cm<br>women<br>$(WC[cm] - 58) \times TG[\frac{mmol}{L}]$	IR – (88)<br>MS – (89)	for IR assessment:<br>>18.1 for women<br>>16.1 for men (88);<br>for MS assessment:<br>>27.57 (89);	- T2D*: better than BMI at identifying adults with diabetes (67)<br>- T2D: may identify MS and IR presence (67,90)<br>- T2D*: the cut-off points for prediabetes and diabetes vary from 21.1 to 35.84 for women and from 30.5 to 56.7 for men, depending on the studies (91–93)
TyG index	Simental-Mendía et al. 2008 (68)	$ln(TG[\frac{mg}{dL}] \times \frac{FPG[\frac{mg}{dL}]}{2})$	IR – lacking data<br>MS – lacking data	for IR assessment:<br>lacking data<br>for MS assessment:<br>lacking data	- higher values in IR (68,74)<br>- the best cut-off point for IR is ln4.65-4.68 (68,94)<br>- the clear cut-off point for MS has not been determined, depending on the ethnicity, MS definition and gender (75,95)<br>- some studies indicate that TyG index has a better predictive ability in identifying IR and MS than HOMA-IR (96,97)<br>- may be an effective tool to predict diabetes* development (98)<br>- may identify MS among blacks (75)<br>- may be a valuable marker in people with normal WC (99)<br>- may predict T2D incidence (100)
VAI	Amato et al.	men	IR – (79)	for IR assessment:	- value = 1 relates to healthy nonobese individuals without adipose

	2010<br>(69)	$\left(\frac{WC[cm]}{39.68+1.88\times BMI}\right)\times\left(\frac{TG\left[\frac{mmol}{L}\right]}{1.03}\right)\times\left(\frac{1.31}{HDL-C\left[\frac{mmol}{L}\right]}\right)$<br><br>women<br>$\left(\frac{WC[cm]}{36.58+1.89\times BMI}\right)\times\left(\frac{TG\left[\frac{mmol}{L}\right]}{0.81}\right)\times\left(\frac{1.52}{HDL-C\left[\frac{mmol}{L}\right]}\right)$	MS – (81,89)	lacking data<br><br>for MS assessment:<br>>1.84 (81);<br>>2.65 (89);	distribution disorders and with normal levels of TG and HDL-C (69)<br>- higher values may indicate visceral fat dysfunction (69)<br>- T1D: higher values may suggest IR presence (79,81)<br>- T1D: positively correlated with insulin requirement (101)<br>- strongly positively associated with cardiometabolic risk (69)<br>- T2D: effective tool to predict prediabetes and diabetes (102,103)<br>- T2D*: better correlated with prediabetes than diabetes (104,105)
TyG-BMI	Er et al.<br>2016<br>(70)	$TyG\ index\times BMI$	IR – lacking data<br>MS – lacking data	for IR assessment:<br>lacking data<br>for MS assessment:<br>lacking data	- higher values in IR (70,74)<br>- they are more efficient than only TyG index (70,104)<br>- TyG-BMI is more efficient in identifying IR than TyG-WC (70)<br>- TyG-WC may be an effective tool to early identify the risks of prediabetes and diabetes* in first-degree relatives of T2D patients (104)<br>- TyG-BMI and TyG-WC may be predictors of diabetes* development in healthy subjects (106)
TyG-WC		$TyG\ index\times WC[cm]$	IR – lacking data<br>MS – lacking data	for IR assessment:<br>lacking data<br>for MS assessment:<br>lacking data	
eIS	Duca et al.<br>2016<br>(71)	individuals with T1D being in fasting state<br>$exp\,(4.1075-0.01299\times WC[cm]-1.05819\times DDI\left[\frac{U}{kg}\right]-0.00354\times TG\left[\frac{mg}{dL}\right]-0.00802\times diastolic\ BP[mmHg])$	IR – (71)<br><br>MS – (81)	for IR assessment:<br>lacking data<br><br>for MS assessment:	- the best fit-model includes the concentrations of adiponectin in the formula and requires being in fasting state (71)<br>- relates to individuals with or without T1D, being or not being in fasting state – depending of the chosen formula (71)

		individuals without T1D being in fasting state $exp\,(7.19138 + 0.10173[men] - 0.01414 \times WC[cm] - 0.33308 \times HbA1c[\%] - 0.01290 \times FPG\left[\frac{mg}{dL}\right] - 0.00316 \times TG\left[\frac{mg}{dL}\right])$		<2.92 generally<br><3.10 for women<br><2.92 for men (81);	- T1D: lower values in IR (71)<br>- T1D: higher values confer protection from the development of albuminuria, diabetic retinopathy and proliferative diabetic retinopathy, as well as the progression of coronary artery calcium score (107)<br>- T1D: the cut-off point for predicting CV disease risk according to the ST1RE is <4.66 (moderate/high risk) and <3.43 (high risk) (86)
lnGDR	Zheng et al. 2017 (72)	$4.964 - (0.121 \times HbA1c[\%]) - (0.012 \times diastolic\ BP[mmHg]) - (1.409 \times WHR)$	IS – (72)<br>MS –(81)	for IR assessment: lacking data<br>for MS assessment:<br><1.8 generally<br><1.81 for women<br><1.77 for men (81);	- T1D: lower values in IR (72)<br>- T1D: can be implemented in individuals with any age onset T1D (72)<br>- T1D: one of the most sensitive and specific method for MS detection (81)
METS-IR	Bello-Chavolla et al. 2018 (73)	$ln(\frac{(2 \times FPG\left[\frac{mg}{dL}\right] + fasting\ TG\left[\frac{mg}{dL}\right]) \times BMI}{ln(HDL-C\left[\frac{mg}{dL}\right])})$	IR – lacking data<br>MS – lacking data	for IR assessment: lacking data<br>for MS assessment: lacking data	- higher values in IR (73)<br>- correlates with visceral, intrahepatic and intrapancreatic fat content (73)<br>- positively associated with prediabetes and T2D (108)<br>- useful for prediction of T2D occurrence (73)<br>- indicator for identifying major adverse cardiac events in diabetic*

					and nondiabetic subjects (108)
TyG-WHtR	Lim et al. 2019 (74)	$\text{TyG index} \times \text{WHtR}$	IR – lacking data<br>MS – lacking data	for IR assessment: lacking data<br>for MS assessment: lacking data	- higher values in IR (74)<br>- predictor of the increased cumulative risk of diabetes* development, better than TyG and other TyG-related parameters (106,109)<br>- T2D: marker for screening fatty liver (110)
TyG-WHpR	Raimi et al. 2021 (75)	$\text{TyG index} \times \text{WHpR}$	IR – lacking data<br>MS – lacking data	for IR assessment: lacking data<br>for MS assessment: lacking data	- higher values in IR (75)<br>- effective in identifying MS (75)
Insulin requirement	(76,77)	$\frac{\text{DDI[U/day]}}{\text{weight[kg]}}$	IR – (76,77)<br>MS – lacking data	for IR assessment: >1 U/kg/day<br>for MS assessment: lacking data	- values >1 U/kg/day indicate insulin resistance in T2D (77), but this value is also used among subjects with T1D in clinical practice
WHtR	-	$\frac{\text{WC[cm]}}{\text{height[cm]}}$	IR – (88)<br>MS – (78,81)	for IR assessment: lacking data<br>for MS assessment: > 0.52 (78,81);	- T1D: higher values in MS (78)<br>- considered as an alternative anthropometric marker for visceral obesity (111)<br>- may be a simpler and more predictive indicator (with the cut-off 0.5) of the “early health risks” which are associated with central obesity than the “matrix” consisting of BMI and WC (112,113)

					- T1D: considered as one of the best anthropometric measures to estimate visceral fat (114)
WC	-	-	IR – (88)<br>MS – (78)	values depending on ethnicity and gender according to IDF (22,78)	- T1D: higher values in patients with MS (78)<br>- T1D: considered as one of the best anthropometric measures to estimate visceral fat (114)
TG/HDL-C ratio	-	$\frac{TG\ [\frac{mg}{dL}]}{HDL-C[\frac{mg}{dL}]}$	IR – (79)<br>MS – (81,89)	for IR assessment:<br>lacking data<br>for MS assessment:<br>>2.0 generally<br>>2.2 in women<br>>2.5 in men (81);<br>>2.18 (89)	- higher values in IR (115)<br>- the cut-off point for IR is ≥3.5 (115)<br>- T2D: the cut-off point for assessing coronary heart disease risk is >1.33 (116)<br>- T1D: it has been proposed that the cut-off points should be lower, as they usually have higher HDL-C because of insulin therapy (79,117)<br>- good predictor for the CV disease and IR development (118,119)<br>- may predict coronary heart disease and CV disease mortality (100)<br>- high values predispose to T2D (100)

*Type of diabetes not specified in the study, likely T2D. Abbreviations: BMI – body mass index; BP - blood pressure; CV – cardiovascular; DDI – daily dose of insulin; eGDR – the estimated glucose disposal rate; eIS – the estimated insulin sensitivity; FPG – fasting plasma glucose; HbA1c/HbA1 – glycated haemoglobin; HDL-C – high density lipoprotein cholesterol; HOMA-IR – the homeostatic model assessment for insulin resistance; HT – hypertension; IDF – International Diabetes Federation; IR – insulin resistance; LAP – the lipid accumulation product; lnGDR – the natural logarithm of glucose disposal rate; METS-IR – the metabolic score for insulin resistance; MS – metabolic syndrome; ST1RE - the Steno type 1 risk engine; T1D – type 1 diabetes; T2D – type 2 diabetes; TG – triglyceride; TG/HDL-C ratio – the triglyceride-high density lipoprotein cholesterol; TyG index – the triglyceride-glucose index; TyG-BMI – the triglyceride-glucose-body

mass index; TyG-WC – the triglyceride-glucose-waist circumference; TyG-WHpR – the triglyceride-glucose-waist-to-hip ratio; TyG-WHtR – the triglyceride-glucose-waist-to-height ratio; U – unit; VAI – the visceral adiposity index; WC – waist circumference; WHR=WHpR – waist-to-hip ratio; WHtR – waist-to-height ratio.
